# Methodological standards in non-inferiority AIDS trials: moving from adherence to compliance

**DOI:** 10.1186/1471-2288-6-46

**Published:** 2006-09-20

**Authors:** Jean-Jacques Parienti, Renaud Verdon, Véronique Massari

**Affiliations:** 1 Inserm UMR-S 707, Paris, F-75012; Université Pierre et Marie Curie-Paris6, UMR-S 707, Paris, F-75012, France; 2 Department of Biostatistics and Clinical Research, Côte de Nacre University hospital, 14033 Caen, France; 3 Department of Infectious Diseases, Côte de Nacre University hospital, 14033 Caen, France

## Abstract

**Background:**

The interpretation of the results of active-control trials regarding the efficacy and safety of a new drug is important for drug registration and following clinical use. It has been suggested that non-inferiority and equivalence studies are not reported with the same quantitative rigor as superiority studies.

**Methods:**

Standard methodological criteria for non-inferiority and equivalence trials including design, analysis and interpretation issues were applied to 18 recently conducted large non-inferiority (15) and equivalence (3) randomized trials in the field of AIDS antiretroviral therapy. We used the continuity-corrected non-inferiority chi-square to test 95% confidence interval treatment difference against the predefined non-inferiority margin.

**Results:**

The pre-specified non-inferiority margin ranged from 10% to 15%. Only 4 studies provided justification for their choice. 39% of the studies (7/18) reported only intent-to-treat (ITT) analysis for the primary endpoint. When on-treatment (OT) and ITT statistical analyses were provided, ITT was favoured over OT for results interpretation for all but one study, inappropriately in this statistical context. All but two of the studies concluded there was "similar" efficacy of the experimental group. However, 9/18 had inconclusive results for non-inferiority.

**Conclusion:**

Conclusions about non-inferiority should be drawn on the basis of the confidence interval analysis of an appropriate primary endpoint, using the predefined criteria for non-inferiority, in both OT and ITT, in compliance with the non-inferiority and equivalence CONSORT statement. We suggest that the use of the non-inferiority chi-square test may provide additional useful information.

## Background

Equivalence and non-inferiority randomized controlled trials are the standard research methodology to demonstrate that a new treatment is equivalent or non-inferior to standard therapy (active-control) in term of efficacy. While an equivalence trial would use the 2-sided 95% confidence interval of the difference between the 2 trial arms, the non-inferiority trial would usually use the 90% confidence interval of the difference, if a 1-sided 5% rather than 2.5% significance test was considered a priori acceptable [1]. Because it is impossible to prove exact equality, the goal in a non-inferiority trial, in situations where the effect compared to placebo is large, is to rule out differences of clinical importance in the primary outcome between the two treatments.

Issues, difficulties and controversies surrounding non-inferiority trials have long been well recognized and extensively reported in many medical settings, including human immunodeficiency virus infection (HIV) [2,3]. Highly active antiretroviral therapy (HAART) delays progression of the acquired immunodeficiency syndrome (AIDS) and increases survival among HIV infected patients. With efficacy rates of 70% [4] and 75% [5] respectively, the space for better antiretroviral agents efficacy has become very tight. However, long term toxicities, pill burden and genotypic resistance call for treatment simplification and alternative new agents. As a consequence, the number of non-inferiority trials has been growing in the recent years in the AIDS therapy literature. Some authors chose to use interchangeably the terms "equivalence" and "non-inferiority", regardless of the hypothesis of the study. Given that the question of interest is not symmetric, we think that they are better described as "non-inferiority" trials[6].

Because efficacy in viral suppression remains the major outcome, new drugs should first prove non-inferiority with respect to prolonged control of HIV replication, as the primary endpoint. Second, the new drugs should provide other advantages. Inevitably, there may have been some tension between marketing purposes and scientific issues in the published reports of those trials. In this paper, our objective was to verify the validity of recently published non-inferiority AIDS trials regarding the primary endpoint.

## Methods

### Study selection and methodological standards

Our aim was to consider a cohort of equivalence or non-inferiority trials published in the area of HIV/AIDS, after HAART became available. We performed a MEDLINE search using the terms equivalence OR non-inferiority AND random* AND HIV (1) and abacavir AND random* (2). 64 (1) and 136 (2) articles were identified. 5 (1) and 5 (2) were selected because they fulfilled the following requirements: randomized controlled clinical trial with 48-week minimum follow-up, initially designed as a non-inferiority or equivalence trial with a prespecified non-inferiority margin, virological primary endpoint and publication in *New England Journal Medicine, JAMA, Lancet, AIDS, Clinical Infectious Diseases, Journal of Infectious Diseases and Journal of Acquired Immune Deficiency Syndrome *between 2001 and 2006. Eight additional articles were identified by examining cross-references or by authors' knowledge of their existence.

We applied traditional methodological requirements for non-inferiority and equivalence trials adapted from Kirshner[7], Jones et al. [8], McAlister and Sackett [9] and Piaggio et al.[1] to eighteen [10-27] active-control trials. We also applied proposed standards in the report of non-inferiority and equivalence trials adapted from Le Henanff [28].

### Statistical analysis

Intent-to-treat (ITT) or on-treatment (OT) analysis 95% confidence interval of the treatment difference were computed using the normal approximation, based on available data included in the flow chart, results section and figures. Two selected studies (ALIZE and SEAL) predefined a 90% confidence interval of the treatment difference, but their conclusions were not affected by the use of the 95% confidence interval (which was used in this paper for homogeneity). Two other selected studies (BMS-045 and CONTEXT) defined the primary endpoint as the log_10 _reduction in HIV viral load, using a time-averaged difference method. For homogeneity with other studies, we considered the more pertinent criteria (closer to the clinical practice) of the percentage of patients with undetectable viral load (< 50 copies/ml or < 400 copies/ml) at week 48 (reported as secondary endpoint).

In case of missing data, the corresponding author of the paper was contacted. When only percentages were available with several possibilities for the numerator due to rounding, we choose on a worst case basis. If original data were censored, we used the cumulative incidence of the primary endpoint in each arm.

Significance testing in establishing non-inferiority between the two arms of a study was computed by the use of the continuity-corrected chi-square of Dunnett and Gent [29] for non-inferiority in intent-to-treat or on-treatment analysis, also on a worst case basis. Briefly, *π*_*1 *_and *π*_*2 *_represent the true proportions of patients with treatment success according to the primary outcome in a random sample of the 2 populations of patients receiving the control treatment and the new drug, respectively. In case of non-inferiority, the expected estimates of *π*_*1 *_and *π*_*2 *_are given by:

π^1=x+y+n2Δn1+n2π^2=x+y−n1Δn1+n2
 MathType@MTEF@5@5@+=feaafiart1ev1aaatCvAUfKttLearuWrP9MDH5MBPbIqV92AaeXatLxBI9gBaebbnrfifHhDYfgasaacH8akY=wiFfYdH8Gipec8Eeeu0xXdbba9frFj0=OqFfea0dXdd9vqai=hGuQ8kuc9pgc9s8qqaq=dirpe0xb9q8qiLsFr0=vr0=vr0dc8meaabaqaciaacaGaaeqabaqabeGadaaakeaafaqabeqacaaabaacciGaf8hWdaNbaKaadaWgaaWcbaGaeGymaedabeaakiabg2da9maalaaabaGaemiEaGNaey4kaSIaemyEaKNaey4kaSIaemOBa42aaSbaaSqaaiabikdaYaqabaGccqqHuoaraeaacqWGUbGBdaWgaaWcbaGaeGymaedabeaakiabgUcaRiabd6gaUnaaBaaaleaacqaIYaGmaeqaaaaaaOqaaiqb=b8aWzaajaWaaSbaaSqaaiabikdaYaqabaGccqGH9aqpdaWcaaqaaiabdIha4jabgUcaRiabdMha5jabgkHiTiabd6gaUnaaBaaaleaacqaIXaqmaeqaaOGaeuiLdqeabaGaemOBa42aaSbaaSqaaiabigdaXaqabaGccqGHRaWkcqWGUbGBdaWgaaWcbaGaeGOmaidabeaaaaaaaaaa@5218@

where *x *and *y *are the observed number of success, *n*_*1 *_and *n*_*2 *_are the 2 sample sizes in the control and the experimental study groups, respectively and Δ the pre-specified margin for non-inferiority.

The continuity-corrected chi-square of Dunnett and Gent [29] (reproduced with written permission) for non-inferiority is given by:

χc2=(|x−x^|−12)2[1x^+1m−x^+1n1−x^+1n2−m+x^]
 MathType@MTEF@5@5@+=feaafiart1ev1aaatCvAUfKttLearuWrP9MDH5MBPbIqV92AaeXatLxBI9gBaebbnrfifHhDYfgasaacH8akY=wiFfYdH8Gipec8Eeeu0xXdbba9frFj0=OqFfea0dXdd9vqai=hGuQ8kuc9pgc9s8qqaq=dirpe0xb9q8qiLsFr0=vr0=vr0dc8meaabaqaciaacaGaaeqabaqabeGadaaakeaaiiGacqWFhpWydaqhaaWcbaGaem4yamgabaGaeGOmaidaaOGaeyypa0JaeiikaGYaaqWaceaacqWG4baEcqGHsislcuWG4baEgaqcaaGaay5bSlaawIa7aiabgkHiTmaalaaabaGaeGymaedabaGaeGOmaidaaiabcMcaPmaaCaaaleqabaGaeGOmaidaaOWaamWaceaadaWcaaqaaiabigdaXaqaaiqbdIha4zaajaaaaiabgUcaRmaalaaabaGaeGymaedabaGaemyBa0MaeyOeI0IafmiEaGNbaKaaaaGaey4kaSYaaSaaaeaacqaIXaqmaeaacqWGUbGBdaWgaaWcbaGaeGymaedabeaakiabgkHiTiqbdIha4zaajaaaaiabgUcaRmaalaaabaGaeGymaedabaGaemOBa42aaSbaaSqaaiabikdaYaqabaGccqGHsislcqWGTbqBcqGHRaWkcuWG4baEgaqcaaaaaiaawUfacaGLDbaaaaa@58FA@

where *m *= *x *+ *y *and x^
 MathType@MTEF@5@5@+=feaafiart1ev1aaatCvAUfKttLearuWrP9MDH5MBPbIqV92AaeXatLxBI9gBaebbnrfifHhDYfgasaacH8akY=wiFfYdH8Gipec8Eeeu0xXdbba9frFj0=OqFfea0dXdd9vqai=hGuQ8kuc9pgc9s8qqaq=dirpe0xb9q8qiLsFr0=vr0=vr0dc8meaabaqaciaacaGaaeqabaqabeGadaaakeaacuWG4baEgaqcaaaa@2E35@ = π^
 MathType@MTEF@5@5@+=feaafiart1ev1aaatCvAUfKttLearuWrP9MDH5MBPbIqV92AaeXatLxBI9gBaebbnrfifHhDYfgasaacH8akY=wiFfYdH8Gipec8Eeeu0xXdbba9frFj0=OqFfea0dXdd9vqai=hGuQ8kuc9pgc9s8qqaq=dirpe0xb9q8qiLsFr0=vr0=vr0dc8meaabaqaciaacaGaaeqabaqabeGadaaakeaaiiGacuWFapaCgaqcaaaa@2E80@_1 _*n*_1_

If Δ is the maximal acceptable difference in success rates between the 2 treatment arms and δ is the observed difference between the experimental and control arms, the equivalence hypothesis can be formulated as pair of one-sided hypothesis:

*H*_01 _: δ ≥ Δ versus *H*_*a*1 _: δ < Δ with a type I error of α_1 _      (1)

and

*H*_02 _: δ ≥ - Δ versus *H*_*a*2 _: δ > - Δ with a type I error of α_2 _      (2)

The type I error probability α for *H*_0 _rejection corresponds to *H*_01 _∪ *H*_02_. Therefore, the P-value for equivalence is the lower chi-square value associated with max (α_1_, α_2_). In a non-inferiority hypothesis, only (1) is necessary. More details have been published elsewhere[30].

**Figure 1:**
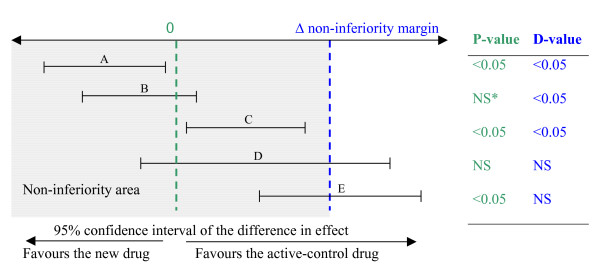
Correspondence between 95% confidence interval of the difference in effect, superiority P-value and non-inferiority D-value. * NS indicates non-significance for superiority or non-inferiority. Case A shows significant superiority of the new drug and necessarily non-inferiority Case B shows significant non-inferiority, but superiority of the new drug is uncertain (inconclusive result) Case C shows both, significant inferiority of the new drug (or superiority of active-control) but nonetheless significant non-inferiority Cases D and E failed to demonstrate non-inferiority (inconclusive result) but E demonstrated significant inferiority (or superiority of active-control).

To avoid confusion between the P-values of superiority tests and the P-values of non-inferiority tests (both are reported in this paper), the latter have been renamed "D-values". When the normal approximation is a valid hypothesis, there is a general consistency between the two-sided 95% confidence interval approach (non-inferiority at α/2 < 2.5%) and the non-inferiority chi-square (D-value < 5%), as shown in Figure 1. D-values and P-values < 0.05 were considered statistically significant.

## Results

### Efficacy of the active control and similar outcome

All of the antiretroviral trials outlined in Table 1 were conducted with active-controls which have previously shown efficacy. 16 studies used a composite endpoint including virologic failure, clinical progression to AIDS or death in compliance with the other new AIDS clinical trials, whereas 2 studies used log_10 _reduction in HIV viral load. However, they reported virologic failure as secondary endpoints.

**Table 1:** Characteristics of the 18 non-inferiority studies

	**Power (%)**	**Double blind**	**Experimental arm(s) (ITT sample size)**	**Control arm (ITT sample size)**
CNAAB3005 [10]	NA	Yes	ABA (262)	PI-based regimen (265)
NEFA [11]	90	No	(a) NVP BID (155)	EFV QD (156)
			(b) ABA BID (149)	
BEST [12]	90	No	IDV/RITO BID (162)	IDV TID (161)
2NN [13]	80	No	(a) NVP QD (220)	EFV (400)
			(b) NVP BID (387)	
			(c) NVP+EFV (209)	
903 [14]	80	Yes	TNF (299)	Stavudine (301)
SOLO [15]	85	No	FPV/RITO QD (322)	Nelfinavir BID (327)
FTC-303 [16]	85	No	FTC QD (294)	3TC BID (146)
EPV20001 [17]	80	Yes	3TC QD (278)	3TC BID (276)
ALIZE [18]	80	No	FTC-ddI-EFV QD (178)	PI-based regimen BID/TID (177)
CNA30024 [19]	85	Yes	ABA BID (324)	AZT BID(325)
BMS-2004 [20]	90	Yes	Atazanavir (405)	EFV (405)
ESS40013 [21]	80	No	Stop EFV (141)	Continue EFV (141)
SEAL [22]	80	No	3TC+ABA QD (130)	3TC+ABA BID (130)
BMS-045 [23]	NA	No	(a) ATA/RITO QD (120)	LOPI/RITO BID (123)
			(b) ATA/SAQUI (115)	
CNA30021 [24]	90	Yes	ABA QD (384)	ABA BID (386)
CONTEXT [25]	NA	No	(a) FPV/RITO QD (105)	LOPI/RITO BID (103)
			(b) FPV/RITO BID (107)	
SHAART [26]	80	No	ABA BID (68)	NVP BID (66)
934 [27]	85	No	TNF+FTC QD (255)	AZT+3TC BID (259)

*one-sided 5% type I error

** one-sided 1.25% type I error

Abbreviations : NA: not available; E: equivalence; NI: non-inferiority; ABA: abacavir PI: protease inhibitor ; NVP: nevirapine ; EFV: efavirenz ; IDV: indinavir ; RITO: ritonavir ; TNF : tenofovir ; FPV: fosamprenavir; FTC : emtricitabine; 3TC: lamivudine ; ddI: didanosine ; AZT : zidovudine ; QD: once-a-day ; BID: twice-a-day ; TID three times-a-day

### Rationale for the non-inferiority margin

**Figure 2:**
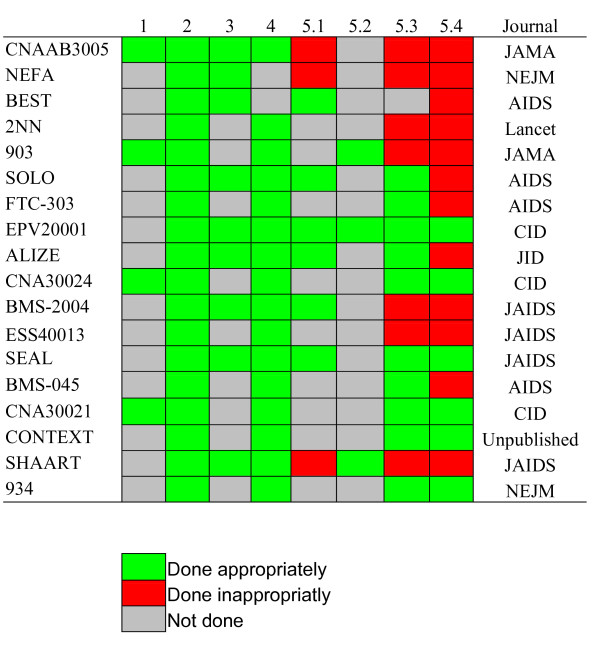
Quality report assessment of non-inferiority trials adapted from Le Henanff et al. [28]. 1. Report the margin and the justification for its choice 2. Appropriate sample size calculation 3. Report both on-treatment and intent-to-treat analysis for the primary endpoint 4. Report 1-sided or 2-sided confidence intervals of treatment difference 5. Conclusion 5.1 Conclude non-inferiority or equivalence only if both ITT and OT analyses permit that or provide separate conclusions. 5.2. Restate the prespecified margin in the abstract 5.3 Make interpretation according to the margin of equivalence or non-inferiority regarding of the primary endpoint 5.4 Conclude with standard and appropriate vocabulary in accordance with the aim and the results of the trial (ie, "non-inferior to" or "equivalent to").

All studies identified a pre-specified non-inferiority margin (criterion for selection). As shown in Figure 2, however, only 4/18 studies reported justification for their choice. In the CNAAB3005 study, the choice of the non-inferiority margin was based on discussion with clinical investigators and with the Food and Drug Administration. The margin of 12% was considered as the largest difference clinically acceptable. In the 903 study, the authors considered that the margin of 10% was a more stringent and conservative non-inferiority criterion. The authors of the CNA30024 commented that it was the appropriate measure for distinguishing the clinical effectiveness of 2 study treatment. Finally, the CNA30024 authors' choice relied on HIV clinicians' judgement as well as on discussion with independent reviewers. Other studies did not comment on their choice, which ranged from 10% to 15% (median: 12%). CONTEXT and BMS-045 considered a non-inferiority margin of -0.5 log_10 _reduction in HIV viral load, without justification. Other issues regarding design are reported in Table 1.

### Confidence interval and superiority testing

All but two trials reported results using the confidence interval approach. In the BEST study, the authors predefined their non-inferiority margin for sample size calculation, but the confidence interval was neither defined nor reported. In the NEFA study, although the confidence interval approach was clearly defined in the statistical analysis section of the article, none was provided in the results section. NEFA, BEST, 2NN, FTC-303, ESS40013 and SHAART studies reported non-significant superiority tests for efficacy to reinforce non-inferiority. The ALIZE and 934 studies switched from the non-inferiority to the superiority hypothesis to declare that the experimental treatment had superior efficacy in the ITT analysis set (for secondary and primary endpoints, respectively), as appropriate.

### Intent-to-treat and on-treatment analysis on the primary endpoint

CNAAB3005, NEFA, SOLO, BEST, EPV20001, ALIZE, BMS-2004, SEAL and SHAART studies (Figure 2) published both ITT and OT analysis (9/18), but only the ALIZE, SOLO, EPV20001 BMS-2004 and SEAL studies found concordant results regarding non-inferiority in the two analysis. The BEST investigators provided separate conclusions for ITT and OT, as appropriate. The ALIZE-trial group conducted ITT, OT and a worst scenario analysis. In CNAAB3005 NEFA and SHAART, the conclusion was based on ITT analysis only. 2NN, FTC-303, EPV20001, ESS40013, CNA30021 and 934 studies described sufficient details to permit alternative analyses, such as OT. We have failed to compute OT analysis from the 903 and CNA30024 studies. Because of the nature of their primary outcome, CONTEXT and BMS-045 studies were not able to provide ITT and OT analysis. Both analysis were provided as secondary endpoints.

### Interpretation and conclusion (non-inferiority margin versus observed upper bound of the 95% confidence interval of the difference)

CNAAB3005 (12% versus 14.3), NEFA (13.5% versus 15.8), 2NN (10% versus 14.0%; 10% versus 14.6%), 903 (10% versus 10.3%) and SHAART (15% versus 17.4%) concluded non-inferiority inappropriately on the basis of their pre-specified margin. In accordance, their non-inferiority D-values were above 5%, as shown in Table 2. BMS-2004 concluded that the two drugs were as efficacious (suggesting equivalence), while the ITT lower bound of the 95% confidence interval (-11.7%) exceeded 10% in favour of the experimental drug. The main BMS-2004 hypothesis (non-inferiority of the experimental drug at 10%) was demonstrated with a D-value = 0.043 (OT analysis). In our analysis of the ESS40013 study (OT), thenon-inferiority margin exceeded the pre-specified non-inferiority margin. Finally, CONTEXT and BMS-045 studies provided a conclusion in accordance with their non-inferiority margin (data not shown).

**Table 2:** Results of the 18 non-inferiority studies

	**Δ Maximum difference % (hypothesis)**	δ (%)*	**UBCI^§ ^of δ (%)**	**Non-inferiority D-value (type of analysis)**	**Superiority P-value (ITT)**	**Abstract authors' conclusion as compared with active-control for primary endpoint**
CNAAB3005	12 (E)	7.3	**14.3**	0.17 (OT)	0.90	Was equivalent to
NEFA	13.5 (NI)					
(a)		3.9	9.8	< 0.001 (ITT)	0.20	No conclusion
(b)		8.5	**15.8**	0.14 (OT)	0.035	A trend toward higher rate of [failure]
BEST	15 (NI)	15.7 (ITT)	**25.9 **(ITT)	1.0 (ITT)	0.003	Superiority of control in ITT
		0.7 (OT)	7.2 (OT)	< 0.001 (OT)	1.0 (OT)	Non-inferiority in OT
2NN	10 (E)					
(a)		5.9	**14.0**	0.36 (ITT)	0.15	Showed similar efficacy
(b)		7.7	**14.6**	0.56 (OT)	0.091**	Showed similar efficacy
(c)		15.4	**23.6**	0.24 (ITT)	0.0003	Did not show efficacy
903	10 (NI)	4.1	**10.3**	0.07 (ITT)	0.19	Highly effective and comparable***
SOLO	12 (NI)	-1.0	6.1	< 0.001 (ITT)	0.78	Provided durable [efficacy]
FTC-303	15 (NI)	5.0	12.9	< 0.01 (ITT)	0.234	Was equivalent to
EPV20001	12 (E)	-0.1	3.6	< 0.001 (OT)	0.81	Regimens were equivalent
ALIZE	15 (NI*)	-2.9	3.6	< 0.001 (ITT)	0.39	Associated with sustained [efficacy]
CNA30024	12 (NI)	-0.8	-6.3	< 0.001 (ITT)	0.82	Not inferior to
BMS-2004	10 (NI)	3.9	9.7	0.043 (OT)	0.16	As efficacious as
ESS40013	12 (NI)	6.1	**13.5**	0.09 (OT)	0.77	Maintained [efficacy]
SEAL	12 (NI*)	-1.5	4.3	< 0.001 (ITT)	0.61	Not inferior to
BMS-045	NA (NI)					
(a)		8.0***	20.4***	NA (ITT)	0.21	As effective as
(b)		19.5***	32.2***	NA (ITT)	0.003	Efficacy was lower than
CNA30021	12 (NI)	2.2	6.6	< 0.001 (OT)	0.61	Not inferior to
CONTEXT	NA (NI)					
(a)		14.0***	28.0***	NA (OT)	0.07	Not shown to be as effective as
(b)		10.0***	23.0***	NA (ITT)	0.30	Not shown to be as effective as
SHAART	15 (NI)	2.1	**17.4**	0.13 (ITT)	0.784	Not inferior to
934	13 (NI)	-3.5	0.5	< 0.001 (OT)	< 0.005	Fulfilled criteria for non-inferiority and proved superior

^§ ^Bold UBCI exceeded the pre-specified non-inferiority margin

*A positive δ corresponds to a higher efficacy of the active-control group, as compared with the experimental group. We choose the OT or ITT in a worst case basis, unless the authors reached separate conclusions for OT and ITT. The numbers may differ from original reports because original reports were stratum-adjusted or used 90% confidence interval.

** If patients who never started treatment were excluded, P = 0.03

***Based on secondary endpoints

Abbreviations: δ : Observed difference between the % of success observed in the control arm minus the % of success observed in the experimental arm ; UBCI : upper bound of the 95% confidence interval; E: equivalence; NI: non-inferiority

BEST, SOLO, FTC-303, EPV20001, ALIZE, CNA30024, SEAL, CNA30021 and 934 conclusions' were appropriate, on the basis of available data.

## Discussion

Trials that assess non-inferiority require rigorous methods for their design, analysis and interpretation. Although the design and the sample size were appropriate for AIDS non-inferiority and equivalence trials, there is room for substantial improvement regarding statistical analysis and interpretation of the results.

Patients with HIV infection would be harmed by deferral of therapy. Consequently, the use of placebo would be unethical [2]. Even if placebo-controlled of HAART therapy are not available, a conclusion about efficacy can be reached because the great majority of patients (about 70%) will not be controlled without treatment [4,5]. Because significant inferiority to active-control would be a major problem for patients, the non-inferiority margin for a new drug should be smaller than the difference between active-control and placebo. Because this effect size is so large, only the clinically chosen margin is really an issue, but is also highly subjective. As a result, this margin varied from the conventional 10% up to 15%. Even the same study group chose different margins in studies 903 (10%) and 934 (13%). A small decrease in margin provides greater assurance of satisfactory effect, but the cost of the study will increase because more patients are required. In the 903-study, the authors could not demonstrate non-inferiority at 10% but they point out in their discussion that this margin was more stringent than the 12% chosen in CNAAB4005. However, if the authors had chosen the less powerful 12% as the maximal limit for non-inferiority, the 95% confidence interval would have been wider, possibly beyond the 12% limit. Consequently, data-driven discussion about the non-inferiority margin after completion of the study is pointless.

Blinding has been described as less efficient in non-inferiority than superiority trials, in particular if the primary endpoint is subjective[31]. For example, a blinded investigator could bias the results toward a preconceived belief in equivalence by assigning similar ratings to the treatment responses of all patients, giving a "bias toward the null". Even when the primary outcome is objective (viral failure, clinical progression or death), however, we believe that blinding is important to protect against bias. Unblinded investigators may provide other effective therapies to patients in the arm that they believe superior or equivalent, such as more regular appointment or adherence support. In addition, patient or physicians may overinterpret subjective endpoints such as side-effects in open-label studies. Finally the absence of blinding can distort the comparability of the groups regarding study withdrawal or patients' adherence, since patients participating in a non-inferiority trial may prefer to receive the simpler therapy. Among the studies observed, significantly more patients discontinued the ALIZE study medication in the control arm for personal reasons, as compared with the simpler, once-a-day experimental group (11% versus 2%, P < 0.0004). This may influence outcome, particularly in an ITT analysis, where withdrawals are considered as failure. Another example comes from the results of the 934 study, where adherence to treatment differed significantly between groups. The conclusion about superior efficacy of the experimental arm in the 934-study may be in part the consequence of greater exposure to the experimental drug. On the other hand, blinding can stand in the way of an optimal drug dispensation in non-inferiority and equivalence trials, in particular if the aim is to simplify antiretroviral therapy. For example, if the purpose is to offer simpler dosage or fewer pills as compared to standard therapy, blinding may require similar regimens in both arms so that any advantages of simplification would be eliminated.

Exclusion of patients after they have been randomized sacrificed the validity of "on-treatment" analysis because it may cause major bias regarding group comparability. For this reason, intention-to-treat analyses has been recognized as the most appropriate and conservative strategy to analyse data of double-blinded trials. However, in case of non-inferiority and equivalence trials, it is well known that this method lacks of robustness since not conservative. For this reason, the study interpretation should also be complemented by "on-treatment analysis"[1,8,9]. If there are discrepancies in the results regarding equivalence or non-inferiority, this should be reported and acknowledged. The CNAAB3005 illustrated how apparent equivalence can be the consequence of a dilutional effect of comparing 2 treatments in the ITT (527 patients) when only 54% of the patients where on-treatment. The same could apply to the ESS40013 study. The use of an "overall" log-rank testing superiority within the 3 arms in the NEFA study may also have blurred the lower efficacy of one study arm, as demonstrated by the "head-to-head" comparison between abacavir and efavirenz.

Like in superiority trials, the choice of the primary outcome is also critical in non-inferiority trials. The BMS-045 illustrated how statistical non-inferiority for viral log difference can be compatible with up to 20.4% of additional virologic failure in the experimental arm, a percentage much larger than non-inferiority margins usually selected for this outcome in this setting.

Finally, the majority of the studies concluded that the effect of at least one experimental arm, based on their prespecified margin, was similar to the control. However, only half of these studies actually demonstrated non-inferiority. Prespecifying the non-inferiority or equivalence margin is necessary but not sufficient to guaranty methodologic quality and appropriate conclusion. We confirmed that AIDS trialists had low adherence to non-inferiority and equivalence methodological standards, as it is the case in other fields[28]. An antiretroviral drug may not prove non-inferiority in term of efficacy but nonetheless be a good alternative because the observed difference is small and the new drug demonstrates better tolerance. This interpretation should, however, be left to the reader. To allow a risk-benefit assessment to be made, the report has a particular obligation to be as clear as possible, using standard statistical vocabulary for non-inferiority and equivalence trials, in compliance with the CONSORT statement.

## Conclusion

Conclusions about non-inferiority should be drawn on the basis of an appropriate confidence interval using a predefined criterion for non-inferiority, shown in both OT and ITT in compliance with the non-inferiority and equivalence extension of the CONSORT statement[1]. We describe how failure to do so will lead to erroneous conclusions. A claim of non-inferiority with a non-inferiority chi-square D-value above 5% is as incorrect as a claim of superiority with traditional null hypothesis testing P-value above 5%. Although the 95% confidence approach is sufficient to reject the null hypothesis, the non-inferiority chi-square provides additional information about the actual degree of significance. Of note, the revised CONSORT statement for superiority trials, item 12a[32] recommends the report of the actual P-values for statistical significance rather than the imprecise threshold "P < 0.05". The additional use of the continuity-corrected non-inferiority chi-square may contribute to avoid misleading interpretation by non-statisticians, for whom significance testing may have a higher impact than confidence intervals. The clinical relevance of the primary outcome on which non-inferiority rely should also be assessed. Reviewers and Editors need to reinforce their standards for acceptance of non-inferiority and equivalence randomized controlled trial. Finally, the importance of critical appraisal has implications for both curricular planning in schools and colleges of medicine, as well as for continuing education programs.

## Competing interests

JJP received research or travel grants from Boehringer Ingelheim, GlaxoSmithKline, Abbott Pharmaceutical, Roche Pharma, GileadSciences. RV received research or travel grants from Bristol-Myers-Squibb, Merck, Boehringer Ingelheim, GlaxoSmithKline, Abbott Pharmaceutical, Roche Pharma, Pfizer and GileadSciences. VM declares that she has no competing interests.

## Authors' contributions

No persons apart from the authors contributed to this paper. JJP had the original idea for the paper. JJP and VM performed the literature search, conducted quality assessment and data extraction and performed statistical analysis. The paper was drafted by JJP and critically appraised for intellectual content by RV and VM, who were also involved in interpretation of the data. All authors read and approved the final manuscript. The guarantor of this paper is JJP.

## Pre-publication history

The pre-publication history for this paper can be accessed here:


